# Analysing Arbitrary Curves from the Line Hough Transform

**DOI:** 10.3390/jimaging6040026

**Published:** 2020-04-23

**Authors:** Donald Bailey, Yuan Chang, Steven Le Moan

**Affiliations:** Centre for Research in Image and Signal Processing, Massey University, Palmerston North 4442, New Zealand; Y.Chang2@massey.ac.nz (Y.C.); S.Lemoan@massey.ac.nz (S.L.M.)

**Keywords:** Hough transform, curve detection, features, edgelets, convex hull, ellipse parameters

## Abstract

The Hough transform is commonly used for detecting linear features within an image. A line is mapped to a peak within parameter space corresponding to the parameters of the line. By analysing the shape of the peak, or peak locus, within parameter space, it is possible to also use the line Hough transform to detect or analyse arbitrary (non-parametric) curves. It is shown that there is a one-to-one relationship between the curve in image space, and the peak locus in parameter space, enabling the complete curve to be reconstructed from its peak locus. In this paper, we determine the patterns of the peak locus for closed curves (including circles and ellipses), linear segments, inflection points, and corners. It is demonstrated that the curve shape can be simplified by ignoring parts of the peak locus. One such simplification is to derive the convex hull of shapes directly from the representation within the Hough transform. It is also demonstrated that the parameters of elliptical blobs can be measured directly from the Hough transform.

## 1. Introduction to the Hough Transform

A common task in many image processing applications is the detection of objects or features (for example lines, circles, etc.). For segmentation, low-level operations detect edge pixels with high intensity gradient, which often correspond to boundaries between regions. It is often then necessary to link these pixels to detect the specific shape corresponding to the object. One popular operation for this is the Hough transform.

The Hough transform was originally proposed for detecting linear features within bubble chamber images [1]. It works by finding support for detected features. The basic principle (for the line Hough transform) is that the line is parameterised, and each detected edge point votes for the set of parameters corresponding to all possible lines which pass through that point (the *voting trace*). When there are many detected points on a line, the parameters of that line will receive many votes from the superposition of the traces generated by each point, indicating significant support for that line. Peaks within the parameter space therefore correspond to clusters of co-linear edge pixels or linear features within an image. The lines are therefore detected by locating the peaks within parameter space. It has been shown [2] that this voting process is a form of maximum likelihood detection.

The main reason for the popularity of the Hough transform is its robustness. This arises because it effectively transforms the problem of shape detection within an image (a global problem) to the detection of local maxima in parameter space (a local problem). Firstly, it is relatively insensitive to noise. Any noise points effectively vote at random locations within parameter space. It requires many co-linear noise points to build a significant peak. The effect of noise is to create a low-level background clutter, from which the significant peaks are usually easily distinguished [3]. Secondly, it is tolerant of missing data, as a result of either occlusion or low contrast. Missing edge points reduce the number of votes within a peak, but as long as there are sufficient edge points detected to form a significant peak, the shape can be detected and reconstructed from the parameters.

The *original Hough transform* is based on a slope-intercept, (m,c), parameterisation:

(1) y=mx+c

with each detected pixel voting on a linear trace in parameter space. Although this is effective for approximately horizontal lines, as the line becomes more vertical, both the slope and intercept tend towards infinity. This was initially overcome by having two parameter spaces, one for horizontal and one for vertical lines [1]. The Hough transform was adapted and made more useable by Duda and Hart [4,5] by choosing a different, (ρ,θ), parameterisation of the lines to give the *standard Hough transform*:

(2) ρ=xcos(θ)+ysin(θ).

The voting trace within parameter space for Equation (2) follows a sinusoidal curve.

This voting approach can easily be extended to detect any parameterised shape, for example circles [4,6] and ellipses. Note that more complex shapes require more parameters, increasing the dimensionality of the parameter space. The voting concept can even be used to detect arbitrary predefined shapes with the *generalised Hough transform* [7], where the arbitrary shape is defined by a template, with parameters defining the shape’s location, orientation and scale. The generalised Hough transform can be considered as a recasting of template matching, made more efficient by only considering edges through the voting process rather than the whole image [8].

Although a curve may be detected with an appropriate parameterisation, or via the generalised Hough transform, this requires prior knowledge of the shape beforehand to set up the parameterisation or shape template [9]. Therefore, if there are several different possible shapes within the image, then the transform must be set up to separately detect each shape. In this paper, we explore the use of the line Hough transform for efficiently detecting and characterising arbitrary curves.

In this context, an *arbitrary curve* is one without necessarily having a pre-defined shape or parameterisation. The role of detecting such a curve is to determine the presence and location of the curve within the image. For a parametric curve, this is a matter of determining the parameters of each instance of the curve within the image. However, detecting an arbitrary curve requires determining the location of local features, which characterise that curve in some way. Associated with detection, is the ability to reconstruct the shape. For parametric curves, the parameters are sufficient to define the curve, enabling reconstruction. For an arbitrary curve, detection implies the ability to reconstruct key features of the curve, or indeed the curve in its entirety.

### 1.1. Limitations of Hough Transform

One limitation of the Hough transform is its computational complexity. This stems from two main sources. Firstly, each detected point will vote for many sets of parameters. This depends on the complexity of the parameterisation and increases with the dimensionality and resolution of the parameter space. It also depends on the resolution of the input image, in particular the number of points detected in the input that contribute to the voting. Secondly, it is necessary to search parameter space to locate the peaks. The complexity of the peak search depends primarily on the volume of parameter space, which again depends on the dimensionality and resolution. Note that for high-dimensional parameter spaces, peak detection is also rendered difficult by the curse of dimensionality. Distances, density and modes are increasingly ill-defined as the number of dimensions (parameters) increases. Many variations of the Hough transform have been proposed to reduce the computational complexity, of which the key principles are listed here.

The storage (and associated voting and peak-finding complexity) can be reduced by reducing the resolution of parameter space. Unfortunately, this also reduces the accuracy of the detected curves. One way around this is to use a hierarchical approach, where a low resolution is used to find initial estimates, and then voting is repeated with a high resolution refinement stage, but limiting the extent of parameter space to small local areas around the initially detected peaks [8].

Reducing the number of points which vote will reduce the complexity of the voting process. The exemplar of this approach is to use only a random selection of detected edge pixels within the voting process [10].

The complexity of voting may be reduced by exploiting edge orientation [6,7,11]. Not every set of parameters on the trace generated by an edge point is equally likely. For example, for line detection, the orientation at each edge point can be detected from the edge gradient. The number of votes cast by an edge pixel can be significantly reduced by only voting for a small range of angles about the detected angle. This *gradient Hough transform* has the added benefit of reducing the clutter within parameter space, making the peaks easier to detect, and can also reduce the occurrence of false peaks.

Reducing the dimensionality of the parameter space reduces the complexity of both voting and finding the peaks. This can be accomplished by a sequence of lower dimensional Hough transforms [12]. For example, for detecting circles, the centre can first be found using a 2D gradient Hough transform followed by a 1D transform for each detected circle centre to determine the most likely radius [13].

Another approach to reducing complexity is to recast the voting process as a form of convolution. This is generally possible when the Hough transform is used for shape detection, and the parameters include the coordinates of the shape within the image [14]. The convolution can then be accelerated by using a fast Fourier transform.

The principle behind all of these approaches is that a single peak in parameter space corresponds to a shape within image space. However, if the shape does not match the parameterised model exactly, then the peak becomes blurred and distorted, because a single set of parameters does not represent the shape. Rather than find a single peak, this paper considers the shape or pattern of the peak, and uses this to directly derive information on arbitrary curves within the image.

### 1.2. Contributions

The novel contributions of this paper are the derivation of a one-to-one mapping between image space and parameter space for the line Hough transform, and to demonstrate its usefulness for efficient detection of arbitrary curves within an image. The remainder of this paper is outlined as follows. Section 2 reviews prior work where more than the peak is used to extract shape information from the parameter space. The mapping between image space and parameter space for the line Hough transform is explored in detail in Section 3, deriving a one-to-one mapping between the two spaces. This mapping is then applied to arbitrary curves, with the characteristics of several pattern classes analysed in Section 4. Finally, Section 5 applies the techniques to complex real shapes (arbitrary curves), and discusses practical issues in applying the techniques.

## 2. Prior Work on Extracting More Information from the Line Hough Transform

Perhaps the most obvious extension of the line Hough transform is for the detection of multiple parallel lines. Parallel lines share a common slope or orientation (*m* in Equation (1) or θ in Equation (2)), enabling them to be easily detected. Under perspective projection, the angle of the lines changes, with parallel lines converging at the vanishing point. In a point-to-line mapping (such as Equation (1) or a parallel coordinate based transformation [15]) the peaks corresponding to a set of parallel lines will also lie on a line within parameter space, enabling the vanishing point and vanishing line to be directly detected [16,17].

The problem of finding and interpreting peaks within a high-dimensional space when detecting arbitrary curves was addressed by Gerig [18]. First, dimensionality is reduced to two parameters, corresponding to the position of the shape within the image. The remaining parameters are effectively projected onto this plane. Then, to prevent ambiguity from such a projection, image space and parameter space are linked, enabling the remaining parameters to be derived directly from the peak. This linking effectively enables the peaks to be interpreted correctly because a peak links directly back to the data that supported it. False peaks (resulting from random coincidental alignment of edge points) can also be rejected from the disparity of the supporting data.

**Figure 1:**
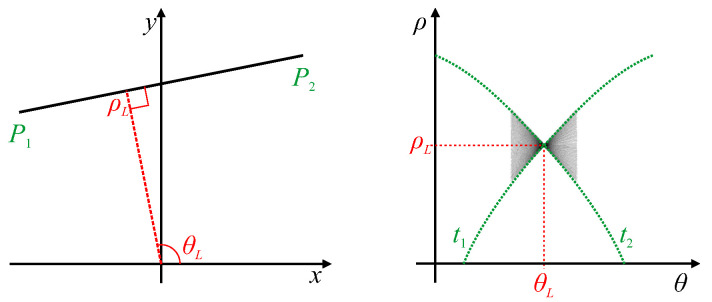
Superposition of traces resulting in a butterfly pattern in the vicinity of a peak. Points $P_{1}$ and $P_{2}$ are the endpoints of a line segment, and generate traces $t_{1}$ and $t_{2}$ which bound the butterfly located at $\left(\theta_{L},\rho_{L}\right)$.

A single peak within parameter space, corresponding to a single line within the image, generally has a *butterfly* shape resulting from superposition of the voting traces (Figure 1). Rather than simply find the peak, Leavers and Boyce [19] devised a matched filter based on the expected butterfly shape to make the peak detection more reliable.

In many applications, the lines do not extend to the edges of the image, and it is often desirable to determine the endpoints of the line segments. Furukawa and Shinagawa [20] did this by analysing the butterfly shape to directly extract the endpoints of the line segments. The traces along the top and bottom edges of the butterfly wings (the curves $t_{1}$ and $t_{2}$ in Figure 1) are generated by the endpoints of the line segment, enabling the endpoints to be determined from these trace orientations. Du et al. [21] built on this concept by showing that the whole butterfly shape contains all of the information associated with the corresponding line segment. By analysing the detailed shape of the butterfly, they were able to derive accurate endpoints even for co-linear segments (which have the same peak parameters) from low-resolution Hough transform parameterisations. For detecting thick lines, rather than use edge detection to get single pixel wide boundaries, Xu et al. [22] used all of the detected points of a thick line for the voting. By measuring the ρ-variance for each θ, and fitting a parabola to the measured variances, they were able to not only detect the endpoints but also estimate the line thickness.

**Figure 2:**
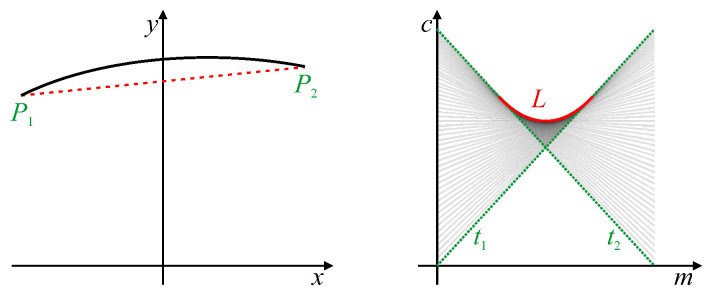
A curved line and its corresponding original Hough transform. Points $P_{1}$ and $P_{2}$ are the endpoints of the curved line, with generated traces $t_{1}$ and $t_{2}$ bounding the butterfly. *L* represents the curved locus of peak votes resulting from changes in slope along the curved line.

Chang et al. [23] used the shape of the peaks within the line Hough transform to directly calibrate lens distortion. With lens distortion, straight lines become curved. This causes the butterfly associated with the peak in parameter space to become smeared because the curved line can no longer be represented by a single set of parameters (see Figure 2). Consequently, the butterfly develops a distinct triangular shape, from which the lens distortion parameters were derived. This work was later extended to fit a parabolic curve to the distributed peak locus [24,25] (the red curve marked *L* in Figure 2). The lens distortion parameters were then derived directly from the parabola coefficients. The original Hough transform was used for this because a parabolic curve in image space maps directly to a parabolic locus [24].

Early work by Leavers and Miller [26] took a similar approach for detecting arbitrary curves. A peak locus is formed within parameter space corresponding to the tangents to the curve in image space, as these will receive a multitude of votes. The peak locus is then detected and used to reconstruct the curve by back-projecting the tangents into image space.

Chang et al. [27] used the line Hough transform to detect circles. A gradient Hough transform focuses the votes along the parameters associated with the curve’s tangent, reducing a lot of the clutter and leaving primarily the peak locus. A circular edge within image space transforms to a sinusoidal peak locus in the parameter space, where the offset of the sinusoid corresponds to the radius, and the amplitude and phase correspond to the position of the circle’s centre within the image in polar coordinates. Detecting the sinusoid’s parameters [28] gives the parameters of the circle.

Leavers [29] took a different approach for the sinusoid detection. The full Hough transform (not the gradient Hough transform) gives two parallel sinusoids for the peak locus. After detecting the peak locus, a second Hough transform is taken to find parallel lines within the locus. The parallel sloping parts of the sinusoids give a pair of stronger peaks from which the spacing between the sinusoids (offset) and phase can be derived. This double Hough transform enables both linear and circular edges to be detected within a single framework.

A different approach was used by Xu et al. [30] for detecting ellipses using a single 2D line Hough transform. Rather than detect the edges of the ellipse, all of the pixels within the ellipse are detected. The elliptical shape is therefore treated as a special case of a thick line, and the statistics of the distribution around a local maximum are used to calculate the best fit ellipse parameters. In essence, this extends their earlier work on thick line detection [22] to determine the ellipse parameters.

All of the prior work assumes that the shape is known beforehand, and information on the parameters of the shape is derived from the shape of the peak in parameter space. However, in the case that the shape is not known, for example when detecting an arbitrary curve, then these methods are not applicable.

## 3. Analysis of Hough Line Transform Mapping

Within this section, we explore the relationship between arbitrary curved edges within an image, and the corresponding representation within the parameter space. The gradient Hough transform is used within this context, because each point on the detected edge is not in isolation, but the set of points along the tangent will vote coherently resulting in a peak (for a straight line) or peak locus (for a curve).

**Figure 3:**
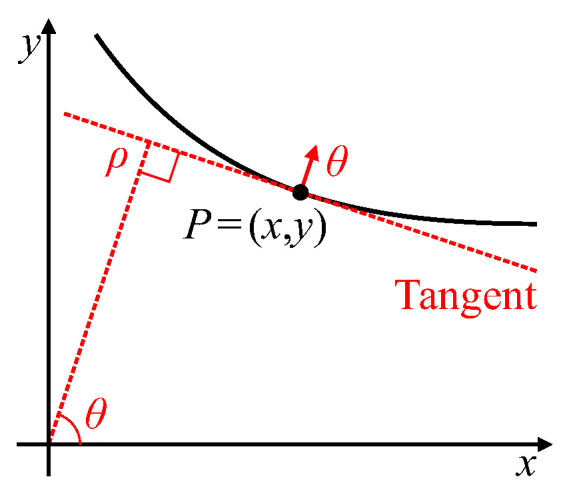
Gradient Hough transform; point (x,y) with gradient θ maps to point (θ,ρ) in parameter space.

Within an image with pixel values I(x,y), edges are typically detected with a gradient operator

(3) $$G\left(x,y\right)=\left(\frac{\partial I}{\partial y},\frac{\partial I}{\partial x}\right),$$

with edges being points of local maximum gradient. Consider a point P=(x,y) on a curved edge, as shown in Figure 3. The gradient direction at that edge point,

(4) θ=tan−1∂I∂y∂I∂x,

will be normal to the tangent at *P*, and thus defines the edge orientation. The gradient Hough transform maps this edgelet, (x,y,θ), to a single point in parameter space, (θ,ρ), using Equation (2).

The converse relationship is also true; although it is well known that a point in parameter space maps to a line in image space (after all, this is the purpose of the Hough transform). However, if the line is not straight, then the feature within parameter space is not a point, but a peak locus. Each point on the locus can be defined by its coordinates and the slope of the locus at that point. Differentiating Equation (2) to get the slope of the locus

(5) $$\frac{d\rho }{d\theta }=-x\sin\left(\theta \right)+y\cos\left(\theta \right)=\rho^{\prime }$$

and solving for *x* and *y* gives

(6) $$\begin{array}{cc} x & =\rho \cos\left(\theta \right)-\rho^{\prime }\sin\left(\theta \right)\\ y & =\rho^{\prime }\cos\left(\theta \right)+\rho \sin\left(\theta \right), \end{array}$$

which is a point on the curve. That is, the triplet within parameter space, $\left(\theta ,\rho ,\rho^{\prime }\right)$, corresponds to a single point on the curve, (x,y), in image space. In other words, these relationships establish a one-to-one mapping between points on a curve in image space, and points on the corresponding peak locus within parameter space. More specifically, the tangents of curves (or edgelets) within the image (defined by (x,y,θ)) effectively map to tangents of the peak locus within parameter space (defined by $\left(\theta ,\rho ,\rho^{\prime }\right)$).

### Extension to Other Parameterisations

The analysis above considered the standard line Hough transform. However, the same analysis can also be applied with any other line Hough transform. The easiest approach is to consider the mappings in terms of distortions within parameter space. Consider the original Hough transform defined by Equation (1). Rearranging Equation (2) gives

(7) $$y=\frac{\rho }{\sin(\theta )}-\frac{x}{\tan(\theta )}$$

with the distortion of the parameter axes given by

(8) $$m=\frac{-1}{\tan(\theta )},\;c=\frac{\rho }{\sin(\theta )}=-\rho \sqrt{m^{2}+1}.$$

Note that typically −1≤m≤1, which implies that $\frac{\pi }{4}\leq \theta \leq \frac{3\pi }{4}$. A similar mapping can be applied to the other quadrant(s).

Since this is a smooth point-to-point mapping from one parameter space to the other, the locus associated with an arbitrary curve will also be mapped smoothly to a locus with the new parameterisation.

For the original Hough transform, a point and its tangent or slope, (x,y,m) maps to a point in parameter space (m,c=y−mx). A point on the parameter locus, (m,c), and its slope $\frac{dc}{dm}=-x$ maps back to the point $\left(-\frac{dc}{dm},c-m\frac{dc}{dm}\right)$ in the image [24].

Since the same principles apply for different parameterisations, the discussion within the next section focuses on the use of the standard Hough transform, except where a different parameterisation simplifies the analysis.

## 4. Mapping of Patterns

Preprocessing the image typically consists of detecting edges using a Canny-like filter (comprised of a small Gaussian smoothing filter to reduce noise and improve the accuracy of detecting the orientation of the gradient; gradient detection with Sobel masks; non-maximal suppression; and thresholding, either with or without hysteresis). From the gradient filters, the gradient orientation, θ, can be determined from Equation (4). If the sign of the edge is important (whether light-to-dark or dark-to-light), then the range of θ needs to be from 0 to 2π. Otherwise, θ can be restricted to the range 0 to π; this can be achieved by wrapping the angle and flipping the locus *L* to account for the change of sign:

(9) $$L_{2}=-L\left(\theta \pm \pi \right).$$

When applying the gradient Hough transform, votes are only cast in a small range around the detected angle (θ±Δθ). This small range reflects the uncertainty in estimating the direction of the gradient [31]. A larger Δθ will result in more reinforcement of the locus in regions of low curvature, as each detected pixel is casting several votes. However, setting Δθ too large will result in a blurring of the locus. In regions of high curvature, a larger Δθ can provide better linking of the locus over a wider change in angle.

### 4.1. Closed Curves

**Figure 4:**
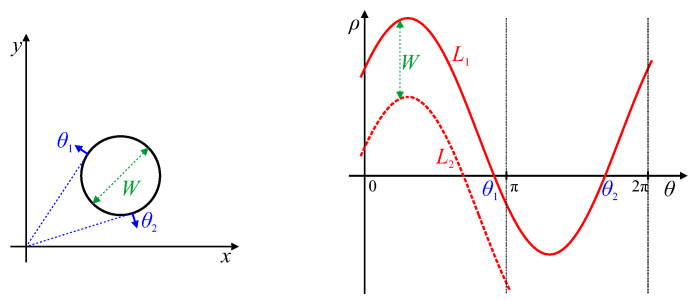
Line Hough transform of a closed curve. The red locus, $L_{1}$, covers the range 0≤θ≤2π. Zero crossings $\theta_{1}$ and $\theta_{2}$ correspond to tangents through the origin. When the sign of the edge is unimportant, the section π≤θ≤2π can be mapped to the range 0≤θ≤π using Equation (9), as shown by the dashed locus, $L_{2}$.

A closed curve will generally result in a peak locus that covers the full range of angles from 0 to 2π, as illustrated by $L_{1}$ in Figure 4. The parameter locus for a convex shape will be a simple periodic curve, as there will be a single tangent for each value of θ. If the sign of the edge is not important, the portion from π≤θ≤2π is shifted by Equation (9) to give the locus $L_{2}$. The locus will pass through ρ=0 when the tangent to the curve passes through the origin. These are marked as $\theta_{1}$ and $\theta_{2}$ in Figure 4. Note that, if the origin (which is usually defined as the centre of the image) is inside the convex closed dcurve, then there will be no zero crossings. The vertical distance between $L_{1}\left(\theta \right)$ and $L_{2}\left(\theta \right)$ corresponds to the width of the object at that angle (Feret or caliper diameter):

(10) $$\begin{array}{cc} W(\theta ) & =\left|L_{1}\left(\theta \right)-L_{2}\left(\theta \right)\right|\\ \; & =\left|L_{1}\left(\theta \right)+L_{1}\left(\theta \pm \pi \right)\right|. \end{array}$$

The absolute value takes into account the orientation of the detected gradient, where the opposite gradient would shift the curve by π and change the sign of ρ. Reversing the edge orientation would effectively swap loci $L_{1}$ and $L_{2}$.

If there are any concavities, then there will either be a corner (see Section 4.4) or an inflection (see Section 4.3), and the locus will double back, having multiple ρ values for a range of θ.

**Figure 5:**
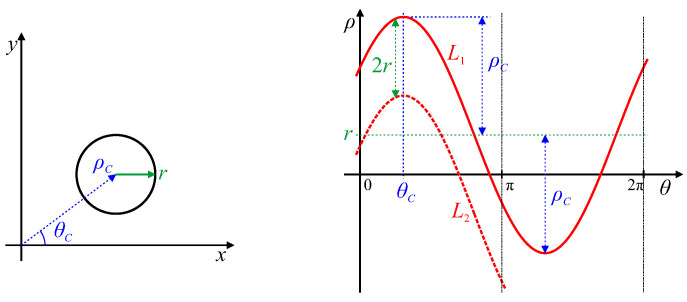
Hough transform of a circular curve of radius *r*. Key features of the parameter locus are marked.

The simplest closed shape is a circle. A circle in an image will result in a sinusoidal locus within parameter space [27,29], as shown in Figure 5. Since the width of the circle (the diameter) is independent of angle, there is a constant offset between loci $L_{1}$ and $L_{2}$. Therefore, the offset of the parameter locus, $L_{1}$, corresponds to the radius of the circle, *r*. The amplitude, $\rho_{C}$, and phase shift, $\theta_{C}$, of the sinusoid correspond to the location of the centre of the circle in polar coordinates as follows. The peak of the locus corresponds to the point on the boundary where the tangent is furthest from the origin. For a circle, this tangent is perpendicular to the line through the centre of the circle, making the origin, circle centre and tangent point co-linear, with the angle of the peak at $\theta_{C}$. As the offset of the sinusoidal locus is *r*, and the amplitude of the peak is $r+\rho_{C}$, the amplitude of the sinusoid is therefore $\rho_{C}$.

**Figure 6:**
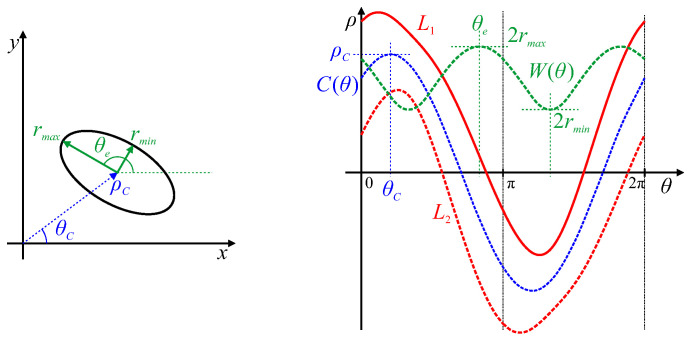
The Hough transform of an elliptical curve generates the parameter locus, $L_{1}$. Constructions from $L_{1}$ are shown for deriving the five ellipse parameters. $L_{2}$ is $L_{1}$ inverted and offset by π. The Feret diameter, W(θ), shown in green, is the difference between $L_{1}$ and $L_{2}$ from Equation (10). The major and minor axes and ellipse orientation, $\theta_{e}$, are derived from this as marked. The centre curve, C(θ), in blue, is the average of $L_{1}$ and $L_{2}$ from Equation (11), with the ellipse centre parameters marked.

A more complex closed shape is an ellipse, which usually requires five parameters: centre position, major and minor axis lengths, and orientation. Since the radius is not constant, the locus is not a sinusoid, but will be stretched or distorted (see $L_{1}$ in Figure 6). The Feret diameter, from Equation (10), will be an offset sinusoid (also distorted slightly) with the offset being the average radius, and the maximum and minimum values of W(θ) corresponding to the lengths of the major and minor axes. The orientation of these axes is given by the θ values where W(θ) is a maximum or minimum respectively (shown in green in Figure 6; for an ellipse, these will be perpendicular). The changing radius of the ellipse makes the centre slightly harder to determine than that of a circle. These can be derived from the curve

(11) $$\begin{array}{cc} C(\theta ) & =\frac{1}{2}\left(L_{1}\left(\theta \right)+L_{2}\left(\theta \right)\right)\\ \; & =\frac{1}{2}\left(L_{1}\left(\theta \right)-L_{1}\left(\theta \pm \pi \right)\right) \end{array}$$

which is the centre between opposite tangents. By symmetry, this corresponds to the point in the centre of the ellipse. Therefore, the Hough transform of this point will also be a sinusoid for an ellipse. The position of the object centre in polar coordinates is given from $\rho_{C}=\max\left(C\left(\theta \right)\right)$ with the corresponding θ giving $\theta_{C}$ (shown in blue in Figure 6).

In general, shifting an object within the image will correspond to adding a sinusoidal curve to the object’s parameter locus. Note, however, that Equation (11) will not be a sinusoid for shapes which are not centro-symmetric, as Equation (11) assumes that the object centre is midway between the two opposite edges However, the peak of Equation (11) will give a unique position within the object that can be used to determine its location (it will be halfway between opposite edges at orientation $\theta_{C}$, where $\theta_{C}$ is the angle where this is furthest from the origin). For an ellipse, the major and minor axes are perpendicular. However, for an arbitrary shaped object, the maximum and minimum of W(θ) are not necessarily perpendicular. In such cases, these features correspond to the major and minor diameters of the shape.

### 4.2. Linear Segments

**Figure 7:**
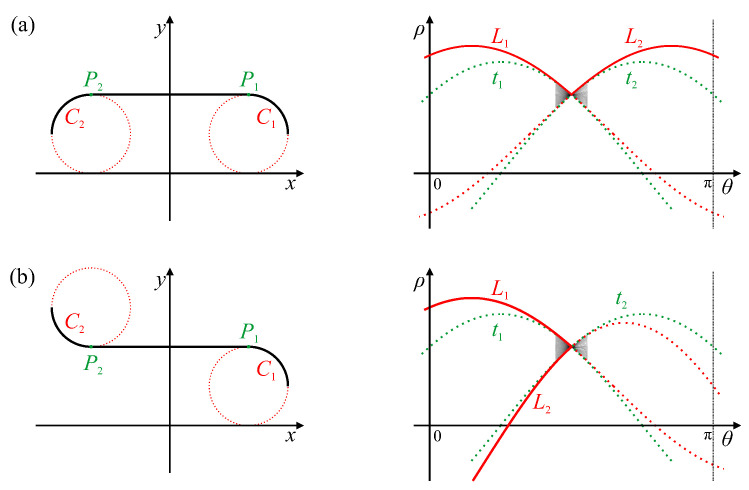
Hough transform of a linear segment with curves at each end. Traces $t_{1}$ and $t_{2}$, generated by segment endpoints $P_{1}$ and $P_{2}$, respectively, bound the butterfly. Circular arcs $C_{1}$ and $C_{2}$ at each end of the line segment generate parameter loci $L_{1}$ and $L_{2}$, respectively. Case (**a**): The slope of the curve continues, thus the loci leave opposite sides of the butterfly. Case (**b**): The slope of the curve changes back, thus the loci leave the same side of the butterfly.

A linear segment will map to a single peak within parameter space. The butterfly shape around the peak represents the range of $\rho^{\prime }$ corresponding to different points along the line segment. The endpoints of the line segment can therefore be derived from the extremes of the range of slopes of the butterfly [20,21], as shown by traces $t_{1}$ and $t_{2}$ in Figure 7. The width of the butterfly will be largely governed by 2Δθ, which is the length along each trace that a detected edge pixel will vote for when using the gradient Hough transform.

If the curve continues smoothly at either end of the linear segment, then the parameters of the linear segment will appear as a butterfly within a peak locus. The locus will leave the point at one of the edge slopes of the butterfly pattern, corresponding to the endpoint of the linear segment. This will result in the peak for the linear segment being a corner point of the peak locus.

If the curvature (the rate of change of the curve’s slope) at the two ends of the linear segment have the same sign, then the peak loci for the curves at the ends of the linear segment will extend from opposite sides of the butterfly, as seen in Figure 7a. However, if the curvature changes sign, then the angle of the tangent, and consequently θ, doubles back, and the peak loci extend from the same side of the butterfly, as demonstrated in Figure 7b.

### 4.3. Inflection Points

**Figure 8:**
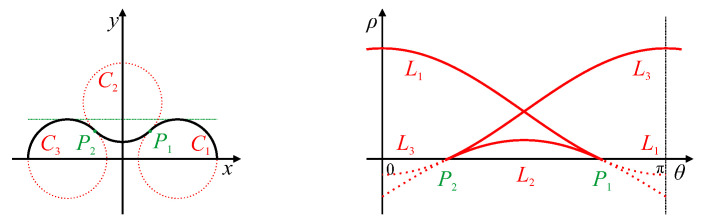
Inflection points $P_{1}$ and $P_{2}$ on the edge result in the locus doubling back with the same slope, generating cusps (labelled $P_{1}$ and $P_{2}$, respectively) in parameter space. Circular arcs $C_{1}$ to $C_{3}$ generate parameter loci $L_{1}$ to $L_{3}$, respectively; the dotted lines in parameter space represent the extension of the loci if the edge continued following the arcs.

A point of inflection occurs when the curvature of the boundary changes sign. This can be considered as a special case of a linear segment with a length of 0. Since the endpoints of the line segment are the same, the corresponding butterfly is degenerate, and the peak locus doubles back at the same angle, resulting in a cusp in the peak locus, as shown in Figure 8. The orientation of the cusp can be used to derive the position of the inflection point along the curve. Note that, although the angle of the cusp is well defined, the precise position of the cusp within parameter space can be harder to determine, because of the blurring along the trace by 2Δθ from the gradient Hough transform.

Let the uncertainty in the angle, θ, of the position of the cusp be δ. Then, from Equation (6), the position of the reconstructed inflection point is

(12) $$\begin{array}{cc} x_{\delta } & =\left(\rho +\delta \rho^{\prime }\right)\cos\left(\theta +\delta \right)-\rho^{\prime }\sin\left(\theta +\delta \right)\\ \; & \approx \left(\rho +\delta \rho^{\prime }\right)\left(\cos(\theta )-\delta \sin(\theta )\right)-\rho^{\prime }\left(\sin(\theta )+\delta \cos(\theta )\right)\\ \; & \approx x-\delta \rho \sin(\theta )\\ y_{\delta } & =\rho^{\prime }\cos\left(\theta +\delta \right)+\left(\rho +\delta \rho^{\prime }\right)\sin\left(\theta +\delta \right)\\ \; & \approx \rho^{\prime }\left(\cos(\theta )-\delta \sin(\theta )\right)+\left(\rho +\delta \rho^{\prime }\right)\left(\sin(\theta )+\delta \cos(\theta )\right)\\ \; & \approx y+\delta \rho \cos(\theta ) \end{array}$$

assuming small δ (i.e., cos(δ)≈1, sin(δ)≈δ, and $\delta^{2}\approx 0$). Equation (12) corresponds to a small uncertainty along the tangent within the image at the point of the inflection. This uncertainty is proportional to δρ, where δ≤Δθ.

### 4.4. Corners

**Figure 9:**
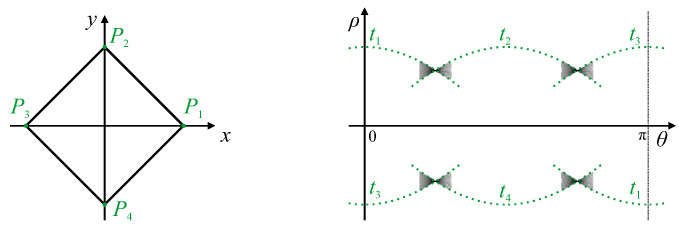
Hough transform of an object with corners. Corner points $P_{1}$ to $P_{4}$ have corresponding traces $t_{1}$ to $t_{4}$. Butterflies at the intersection of traces correspond to the linear segments between corners. Note that, with the gradient Hough transform, the traces between the butterflies will not actually receive any votes; they are drawn here to illustrate the relationship between the bounding edges of the butterflies.

A sharp corner will result in a gap in the peak locus because the slope of the edge changes suddenly at a corner. A polygon with *N* sides would show *N* distinct and separate peaks within parameter space, with a corresponding butterfly at each peak (see Figure 9). Each corner point on the polygon corresponds to the intersection of two linear segments. Therefore, in parameter space, successive peaks will fall on the same sinusoidal voting trace (in the standard Hough transform, or linear trace in Hough’s original transform). This trace will therefore link the butterflies associated with adjacent line segments, as illustrated in Figure 9. Note that, with the gradient Hough transform, there will be very few votes along the trace, thus the peak locus will not necessarily be visible. However, the Gaussian filter used for preprocessing will cause sharp corners to become rounded, and this may result in a few votes along the trace between linear segments, although there are unlikely to be enough votes to result in a distinct peak locus.

**Figure 10:**
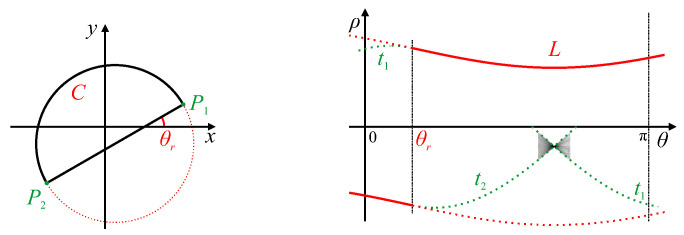
Hough transform of a semi-circle with corners. Corner points $P_{1}$ and $P_{2}$ have corresponding traces $t_{1}$ and $t_{2}$ (shown dotted because the sharp corners will not result in votes with the gradient Hough transform). The peak of the butterfly gives the parameters of the line connecting $P_{1}$ and $P_{2}$. The circular segment, *C*, generates the peak locus, *L* (the dotted segment corresponds to the locus of the truncated circular segment in the image).

Another example is given in Figure 10. The semicircular shape produces a peak locus, marked *L*. This locus terminates at the parameters corresponding to the corners $P_{1}$ and $P_{2}$ because of the discontinuity of the angle of the curve at these corner points. If the locus did continue, it would follow the traces $t_{1}$ and $t_{2}$, respectively, as these represent the tangents at different angles going around the corners. Since $P_{1}$ and $P_{2}$ are joined by a straight line, there will be a single peak (and corresponding butterfly) bounded by traces $t_{1}$ and $t_{2}$.

**Figure 11:**
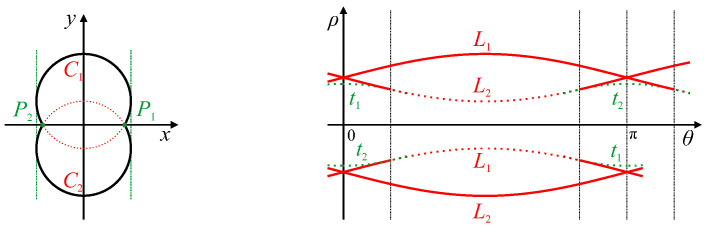
Hough transform of two intersecting circles. The circular arcs $C_{1}$ and $C_{2}$ map to the peak loci $L_{1}$ and $L_{2}$, respectively (with the dotted segments of *L* corresponding to the dotted segments of *C*). Corner points $P_{1}$ and $P_{2}$ have corresponding traces $t_{1}$ and $t_{2}$ (shown dotted because they will receive no votes in the gradient Hough transform).

A more complex example with a pair of concave corners is shown in Figure 11. The peak locus for each circular section spans an angle of $\frac{4\pi }{3}$. The angle of the corners means θ goes back by $\frac{\pi }{3}$, asshown by traces $t_{1}$ and $t_{2}$. The peak loci intersect at angles θ=0 and θ=π, corresponding to the left and right edges of the pattern where the curved sections share the same tangent, as shown by the green dotted lines in the left panel of Figure 11. The different slopes of the loci at their intersection correspond to the different points in the image where the tangents are co-linear.

### 4.5. Convex Hull

The convex hull of a shape may be obtained directly from its Hough transform. If the object is convex, then the peak locus already represents the convex hull.

However, if the shape has concavities, there will be regions of the boundary with negative curvature, with corresponding inflections in the boundary. As shown in Section 4.3, such inflections cause the locus to double back. Any concavity will have an even number of such inflections, with the peak locus crossing at the parameters representing the common tangents to the different parts of the curve. The outermost of these common tangents therefore represents the convex perimeter of the associated concavities. The corresponding peak locus intersection is named a *convex intersection*.

Therefore, depending on the edge sense (light-to-dark or dark-to-light), either the maximum or minimum boundary of the locus represents the convex hull of the object shape. (If the range of θ was mapped to 0 to π, then both the minimum and maximum boundaries are used.)

**Figure 12:**
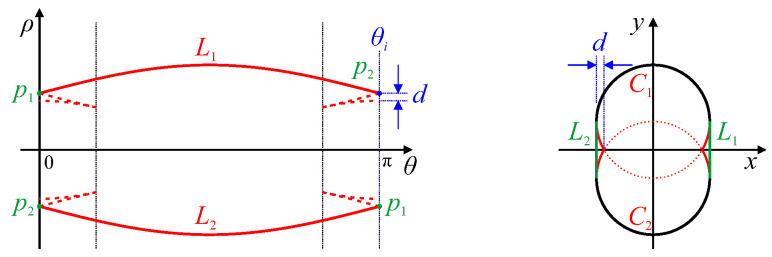
Selecting only the maximum and minimum locus for each angle gives the convex hull of the shape. Convex intersection points $p_{1}$ and $p_{2}$ correspond to lines $L_{1}$ and $L_{2}$ within the image which are the convex edges that span the concavities. The depth of the concavity is *d*.

This can be demonstrated for the intersecting circles of Figure 11 in Figure 12. Removing the cusps will remove the concavities, and the convex intersections correspond to the convex edges in the image which span the concavities. The slopes of the peak locus at such a convex intersection correspond to the vertices at each end of the convex edge line segment. Filling in the concavity in this way effectively replaces the cusps associated with the concavity with a butterfly at the convex intersection.

The maximum depth of the concavity can also be directly measured from the peak locus. Let the concavity depth be defined as the maximum distance within the concavity measured perpendicular to the convex edge. Let $\theta_{i}$ be the angle of the convex intersection of the peak locus; this corresponds to the perpendicular to the convex edge. All lines at this angle will be parallel to the convex edge. The point on the original locus furthest from the convex intersection point will lie on the parallel line that is furthest from the convex edge, i.e., the point at angle $\theta_{i}$ within the concavity that is furthest from the convex intersection within parameter space. This is illustrated in Figure 12.

### 4.6. Parabolas

For curves that can be represented as a parabola, i.e.,

(13) $$y=a_{2}x^{2}+a_{1}x+a_{0}$$

(or similarly $x=a_{2}y^{2}+a_{1}y+a_{0}$), the peak locus can be represented as a parabola when using the original Hough transform [24]. From Equations (1) and (13), the parameters of the peak locus can be represented parametrically (as a function of *x*) as

(14) $$\begin{array}{cc} m & =\frac{dy}{dx}=2a_{2}x+a_{1}\\ c & =y-mx=a_{0}-a_{2}x^{2}. \end{array}$$

**Figure 13:**
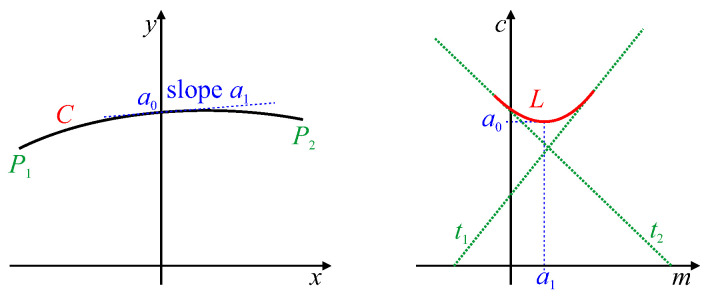
A parabolic curve, *C*, and its corresponding peak locus, *L*, in the original Hough transform. Traces $t_{1}$ and $t_{2}$ are generated from endpoints $P_{1}$ and $P_{2}$, respectively (these may be the edges of the image). Parabola parameters $a_{0}$ and $a_{1}$ map directly to features of the locus. The quadratic parameter, $a_{2}$, of *C* is also reflected directly in the quadratic parameter of *L*.

Then, eliminating *x* gives

(15) $$\begin{array}{cc} c & =a_{0}-\frac{{\left(m-a_{1}\right)}^{2}}{4a_{2}}\\ \; & =\frac{-1}{4a_{2}}m^{2}+\frac{a_{1}}{2a_{2}}m+\left(a_{0}-\frac{a_{1}^{2}}{4a_{2}}\right)\\ \; & =p_{2}m^{2}+p_{1}m+p_{0}, \end{array}$$

which is also a parabola in parameter space. The parabola coefficients have a direct mapping within the Hough transform as demonstrated in Figure 13. The inverse relationship,

(16) $$\begin{array}{cc} \frac{dc}{dm} & =-x=2p_{2}m+p_{1}\\ y & =mx+c=p_{0}-p_{2}m^{2}\\ \; & =p_{0}-\frac{{\left(x+p_{1}\right)}^{2}}{4p_{2}}, \end{array}$$

also has a direct mapping between domains, and was used by Chang [25] for modelling and calibrating lens distortion in images.

## 5. Testing on a Real Image

The preceding analysis assumed that the image and transform data were continuous. In practise, the situation is more complicated since both the image and parameter space are spatially quantised (sampled). In particular, the estimates of slopes or gradient orientations are less accurate, particularly of the peak locus.

To explore the ideas presented in the previous section, algorithms were developed in Matlab under Windows. These algorithms are represented within this section in pseudocode form.

### 5.1. Hough Transform

**Figure 14:**
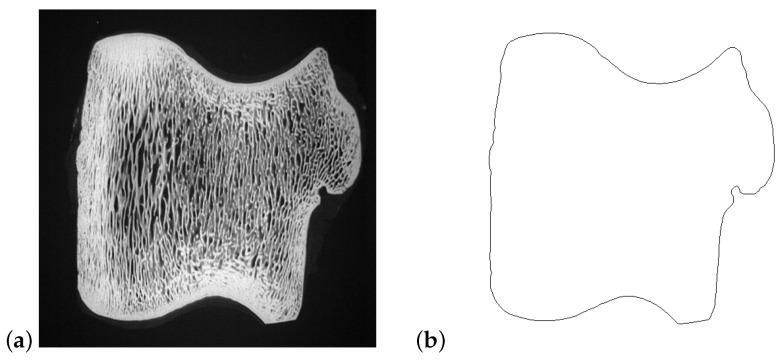
Example of processing an image with a complex shape: (**a**) the image; and (**b**) outline of the image detected by the Canny filter.

To test the pattern generation and reconstruction, the line Hough transform was applied to a complex shape, as shown in Figure 14a. This image is 512×512 pixels, with the origin in image space defined as pixel (256,256). For detecting the edges, the following parameters were used for the Canny filter: Gaussian smoothing $\sigma =\sqrt{2}$ and thresholds [0.2,0.5]. The outside edge detected by the Canny filter is shown in Figure 14b.

**Figure 15:**
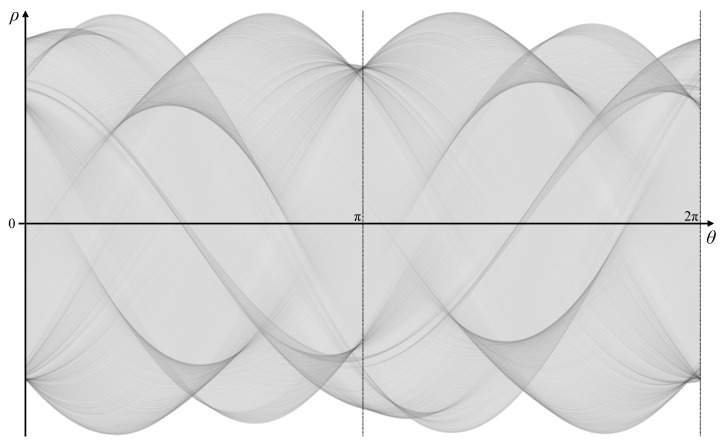
The full Hough transform of the boundary from Figure 14 (contrast enhanced for visibility).

Since the most distant point in the image from the origin has $\rho =256\sqrt{2}=362$, the accumulator in parameter space must extend to −362≤ρ≤362. Because of the complexity of the shape, the full angle range from 0≤θ<2π was used. An angle resolution of $\theta_{\Delta }=0.0044$ radians (0.25°) was chosen to simplify the tracking of the peak locus (so that the maximum slope of the peak locus is less than 1 enabling a 3×3 window to be used for calculating $\rho^{\prime }$; the worst case would require $\theta_{\Delta }<\frac{1}{362}=0.0028$ for a tangent in the very corner of the image passing through the origin). This gives a 724×1440 accumulator image. Algorithm 1 calculates the full Hough transform, and, when applied to this detected edge, the parameter space is shown in Figure 15. Although the main peak loci can be seen, the minor ones are hidden within the background clutter.
**Algorithm 1** Full Hough transform.**Input:** Binary image I[x,y] after Canny filtering (origin in centre of image)**Output:** Vote image V[θ,ρ]
1:**for all**(ρ,θ)**do**2: V[ρ,θ]=0           ▹Clear vote accumulators3:**end for**4:**for all**(x,y)**where**I[x,y]=1**do**   ▹Detected pixels5: **for** θ=0 **to** 2π **do**6:  ρ=xcos(θ)+ysin(θ)   ▹Equation(2)7:  V[θ,round(ρ)]+=1      ▹Accumulate votes8: **end for**9:**end for**

Since the Canny filter also provides edge orientation, this can be used with the gradient Hough transform to reduce the clutter, making the loci clearer to see and interpret. For this, Δθ=±0.035(±2°). This was chosen based on the estimation of the uncertainty of edge orientation detection by the Canny filter. It also balances between reinforcing and blurring the peak locus where the edge angle changes slowly, and not losing too many points when the edge angle changes rapidly. Simply using Equation (2) to vote for the nearest ρ bin means that it is very difficult to measure $\rho^{\prime }$ with any level of accuracy (because of the sampling within parameter space) without fitting a curve over a large number of pixels. To overcome this limitation, the fractional part of ρ as calculated by Equation (2) is used to split the vote proportionally between two adjacent ρ bins. Let $\rho_{i}\left(\theta \right)$ and $\rho_{f}\left(\theta \right)$ be the integer and fractional components of the result of Equation (2) for a given pixel and value of θ. Votes are then castas

(17) $$\begin{array}{cc} V[\theta ,\rho_{i}\left(\theta \right)] & \;+\;\;=\;1-\rho_{f}\left(\theta \right)\\ V[\theta ,\rho_{i}\left(\theta \right)\;+\;1] & \;+\;\;=\;\rho_{f}\left(\theta \right). \end{array}$$

The corresponding gradient Hough transform is computed using Algorithm 2.
**Algorithm 2** Gradient Hough transform.**Input:** Binary image I[x,y] after Canny filtering (origin in centre of image)
**Input:** Orientation image θ[x,y] of detected pixels from the Canny filter**Output:** Vote image V[θ,ρ]1:**for all**(ρ,θ)**do**2: V[ρ,θ]=0               ▹Clear vote accumulators3:**end for**4:**for all**(x,y)**where**I[x,y]=1**do**      ▹Detected pixels5: **for** θ=θ[x,y]−Δθ **to** θ[x,y]+Δθ **do**  ▹Vote only for a small range of angles6:  ρ=xcos(θ)+ysin(θ)       ▹Equation (2)7:  $\rho_{i}=\left\lfloor \rho \right\rfloor$                ▹Separate integer and fractional components8:  $\rho_{f}=\rho -\rho_{i}$9:  $V\left[\theta ,\rho_{i}\right]\;+\;\;=\;1-\rho_{f}$         ▹Distribute accumulated votes10:  $V\left[\theta ,\rho_{i}\;+\;1\right]\;+\;\;=\;\rho_{f}$         ▹between adjacent bins, Equation (17)11: **end for**12:**end for**

**Figure 16:**
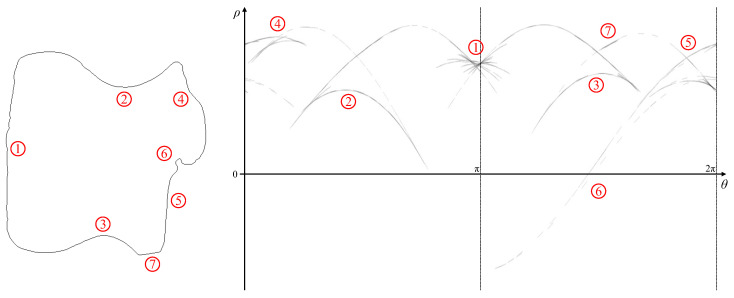
The shape and its pattern in parameter space after the gradient Hough transform (contrast enhanced for visibility). Numbered features link the patterns with the corresponding regions on the object shape.

Distributing the votes in this way enables the peak locus to be measured to sub-pixel accuracy, enabling $\rho^{\prime }$ to be calculated using only adjacent θ bins. The corresponding gradient Hough transform is illustrated in Figure 16.

### 5.2. Locus Analysis

Before looking at reconstructing the boundary, several observations can be made from the patterns within parameter space.

1. Even minor noise on the boundary can result in inflections. In some cases, this leads to a broadening of the peak locus, reflecting the uncertainty in the actual edge position. In other cases, there is a series of distinct cusps. These are most noticeable in this example around ➀ corresponding to the ripples in the left edge of the image, where at least 14 distinct cusps may be found.
2. Cusps come in pairs, one pointing to the left and one pointing to the right. This can most easily be seen for the large concavities on the top ➁ and bottom ➂ of the object. Within a series of cusps (for example, ➀), the locus corresponding to following around the object boundary will have alternating left and right cusps. For generally increasing θ, the left cusps are in sequence from top to bottom, and the right cusps from bottom to top.
3. The number of minor cusps may be reduced by increasing the smoothing of the Gaussian filter as part of the Canny edge detector (by increasing σ).
4. Alternatively, the weaker cusps in the pattern may simply be ignored. The weaker cusps have less support (fewer pixels have voted for these parameters), thus ignoring those pixels will remove the concavities. In general, ignoring any features within parameter space will simplify the curve.
5. In particular, the locus following the maximum value of ρ for each θ corresponds to the convex hull of the shape. (Note that, for a dark object on a light background, the minimum value of ρ(θ) should be used.) The concavities on the top right and bottom right edges are represented by features ➃ and ➄, respectively.
6. Where the curvature changes rapidly (for example, at sharp corners or in the circular concavity on the right side of the shape ➅), the locus is incomplete and appears as dashed lines. Each dash represents the votes from one pixel, with the length of the dash along the θ axis being 2Δθ.
7. The thickening of the line at ➆ is actually a small butterfly corresponding to the short line segment within the image. However, the segment is short (especially since the ends are rounded slightly with Gaussian smoothing) and there is insufficient resolution on the ρ axis to clearly see the butterfly shape.

**Figure 17:**
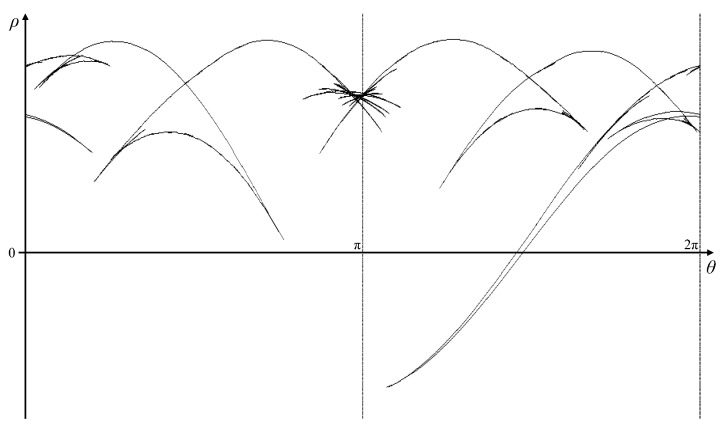
The underlying peak locus from the Hough transform in Figure 16 (obtained using Algorithm 3, not derived from the previous figure).

The underlying peak locus from Figure 16 is shown in Figure 17 (obtained using Algorithm 3). Note that this was not constructed by voting, but by following the boundary in the original image, mapping the individual pixels to the nearest set of parameters. Δθ was adjusted dynamically so that there was no overlap between neighbouring pixels within parameter space. This effectively gives a continuous path within parameter space, with each bin (set of parameters) receiving only one vote, even where the paths overlap. This is provided for visualisation only, and is not suitable for actual reconstruction because $\rho^{\prime }$ cannot be accurately measured from this.
**Algorithm 3** Shape of underlying peak locus.**Input:** 1D array, *B*, containing the sequence of detected boundary pixels (x,y)
**Input:** Orientation image θ[x,y] of detected pixels from the Canny filter
**Output:** Vote image L[θ,ρ]1:**for all**(ρ,θ)**do**2: L[ρ,θ]=0                             ▹Clear locus image3:**end for**4:**for**n=1**to** length (B) **do**                      ▹Detected pixels in sequence5: **for** $\theta =\frac{1}{2}\left(\theta \left[B\left[n\;-\;1\right]\right]\;+\;\theta \left[B\left[n\right]\right]\right)$ **to** $\frac{1}{2}\left(\theta \left[B\left[n\right]\right]\;+\;\theta \left[B\left[n\;+\;1\right]\right]\right)$ **do**  ▹Link adjacent detected pixels6:  ρ=xcos(θ)+ysin(θ)                     ▹Equation (2)7:  L[θ,round(ρ)]=1                      ▹Mark underlying locus points8: **end for**9:**end for**

### 5.3. Curve Reconstruction

To reconstruct the shape, the locus was detected within Figure 16 by finding the cells with a local maximum of votes (see Algorithm 4). For each column of the accumulator matrix (i.e., each θ value), the ρ value of each local maximum was determined. To estimate ρ to the sub-pixel level, linear interpolation between the maximum and the maximum of its adjacent neighbours was used. Let $V[\rho_{m}]$ and $V[\rho_{m}+1]$ be the votes of the two adjacent bins (indices $\rho_{m}$ and $\rho_{m}+1$) for a given local maximum. The sub-pixel maximum, $\rho_{M}$, is then estimated as

(18) $$\rho_{M}=\rho_{m}+\frac{V[\rho_{m}+1]}{V\left[\rho_{m}\right]+V\left[\rho_{m}+1\right]}.$$

**Figure 18:**
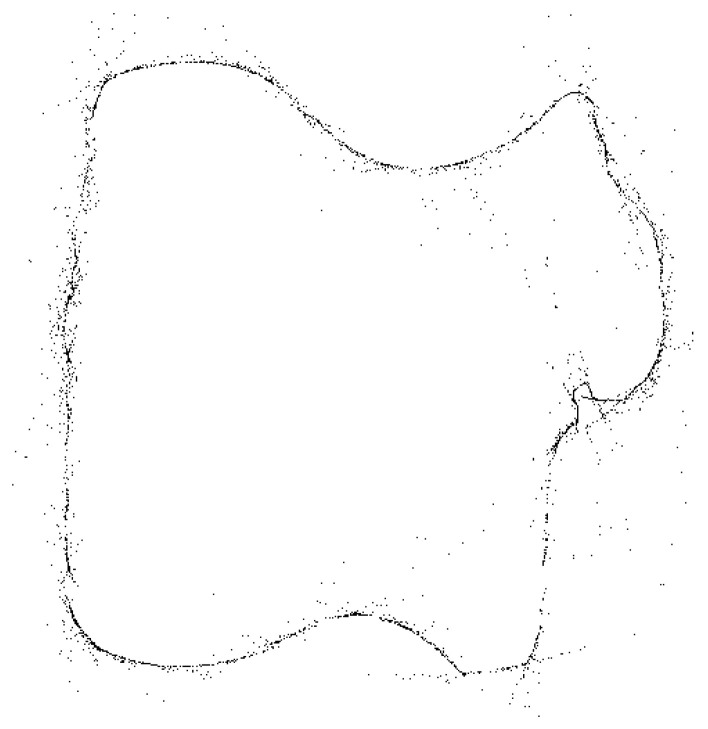
Simple reconstruction of the boundary points (using Algorithm 4).

This sub-pixel estimate then enables the slope of the locus at this point, $\rho_{M}^{\prime }$, to be found by taking the central difference between the locations of sub-pixel maxima on opposite sides of a 3×3 window:

(19) $$\rho_{M}^{\prime }\left(\theta_{i}\right)=\frac{\rho_{M}\left(\theta_{i}+1\right)-\rho_{M}\left(\theta_{i}-1\right)}{2\theta_{\Delta }}$$

where $\theta_{i}$ is the column number in the accumulator array and $\theta_{\Delta }$ is the column spacing. The image coordinates are then calculated for each local maximum using Equation (6), rounding the coordinates to the nearest pixel. The resulting reconstruction is shown in Figure 18. While many of the points are reconstructed accurately (they coincide with the original points), there is also a cloud of noise pixels around the edge. These result primarily from errors in estimating $\rho^{\prime }$ leading to the selection of an incorrect point along the tangent. There are two causes for these errors. Firstly, Equation (19) has a small number in the denominator, so any errors in estimating the position of the peak locus get amplified. Secondly, where there are multiple peak loci in close proximity, the resolution of parameter space means that these are in the same or adjacent bins (for example around intersections or near cusps). The votes for these loci are combined, distorting the estimate of $\rho^{\prime }$. As the loci separate, the wrong adjacent bins can be selected for Equation (19), leading to outliers. Since each detected point within parameter space is being used to construct only a single point within image space, there are also gaps, particularly within straight lines, where the butterfly pattern should be used to reconstruct a line segment rather than a single point.

**Figure 19:**
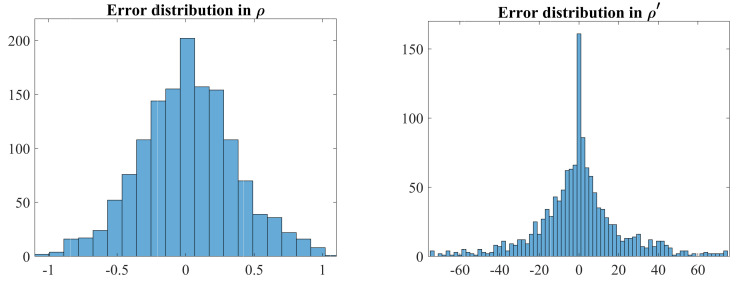
Error distributions of measured values of ρ and $\rho^{\prime }$ from the Hough transform. Standard deviations are $\sigma_{\rho }=0.35$ pixels and $\sigma_{\rho^{\prime }}=21.4$ pixels/radian.

The error distributions of the measured values of ρ and $\rho^{\prime }$ were determined by comparing the data used for voting (from applying Equations (2) and (5) to the original pixels) with that derived from the corresponding detected point on the peak locus (using Equations (18) and (19)). The resulting error distributions are given in Figure 19. Note that there is some uncertainty in these data as the θ values derived from the Canny filter are also noisy. However, it does provide an indication of the uncertainty in the data extracted from the Hough transform. The errors in ρ are a fraction of a pixel (standard deviation $\sigma_{\rho }=0.35$ pixels), as expected, and follow a normal distribution. This shows that the procedure for distributing the votes between adjacent ρ bins does not appear to introduce a bias (mean $\mu_{\rho }=0.017$ pixels), and successfully enables sub-pixel estimation of ρ. However, theerrors in $\rho^{\prime }$ are significantly larger (standard deviation $\sigma_{\rho^{\prime }}=21.4$ pixels/radian), and, apart from the peak around 0 pixels error, also appear to follow a normal distribution. These larger errors are a result of division by $\theta_{\Delta }$ in Equation (19). There are also a number of outliers (the distribution tapers off more slowly than expected outside ±40 pixels/radian); closer investigation reveals that these outliers result from the simple approach used to estimate peak position to sub-pixel accuracy, and occur primarily where multiple loci intersect or interact. The slope estimation appears to be unbiased (mean $\mu_{\rho^{\prime }}=-0.026$ pixels/radian), but the large spread is the primary cause for the noise observed in Figure 18.
**Algorithm 4** Simple reconstruction.**Input:** Vote image V[θ,ρ] from gradient Hough transform**Output:** Reconstructed image R[x,y]1:**for all**(x,y)**do**2: R[x,y]=0                   ▹Clear reconstruction image3:**end for**4:**for each**θ**do**5: Find local maxima (peak locus), $\rho_{m}$         ▹$V\left[\theta ,\rho_{m}\;-\;1\right]\leq V\left[\theta ,\rho_{m}\right]\geq V\left[\theta ,\rho_{m}\;+\;1\right]$6: **if** $V\left[\theta ,\rho_{m}\;-\;1\right]>V\left[\theta ,\rho_{m}\;+\;1\right]$ **then**7:  $\rho_{m}\leftarrow \rho_{m}-1$                 ▹So that peak is between $\rho_{m}$ and $\rho_{m}\;+\;1$8: **end if**9: Calculate peak to sub-pixel accuracy, $\rho_{M}$      ▹Equation (18)10:**end for**11:Calculate slope of locus at local maxima, $\rho_{M}^{\prime }$     ▹Equation (19)12:**for all** detected local maxima **do**13: Calculate image coordinates, (x,y)        ▹Equation (6)14: R[round(x),round(y)]=1         ▹Mark detected points15:**end for**

### 5.4. Filtered Reconstruction

An improved reconstruction may be obtained by first linking the boundary pixels to fill in gaps (to give a continuous boundary), and then filtering to remove duplicates and outlier pixels (see Algorithm 5). In calculating $\rho^{\prime }$, peaks in adjacent θ bins are used. Linking these adjacent peaks creates chains or segments along the peak locus. Inevitably, there are gaps within these chains, firstly, where the curvature changes rapidly at corners, and, secondly, where a peak locus is intersected by a stronger peak locus. These peak locus segments are sorted and linked based on their proximity in both parameter space and their mapped location in image space. Note that the parameter loci of concave segments need to be reversed.
**Algorithm 5** Improved reconstruction through linking points and filtering.**Input:** Vote image V[θ,ρ] from gradient Hough transform**Output:** Reconstructed image R[x,y]1:**for all**(x,y)**do**2: R[x,y]=0                 ▹Clear reconstruction image3:**end for**4:**for each**θ**do**5: Find local maxima (peak locus), $\rho_{m}$      ▹$V\left[\theta ,\rho_{m}\;-\;1\right]\leq V\left[\theta ,\rho_{m}\right]\geq V\left[\theta ,\rho_{m}\;+\;1\right]$6: **if** $V\left[\theta ,\rho_{m}\;-\;1\right]>V\left[\theta ,\rho_{m}\;+\;1\right]$ **then**7:  $\rho_{m}\leftarrow \rho_{m}-1$              ▹Peak is between $\rho_{m}$ and $\rho_{m}\;+\;1$8: **end if**9: Calculate peak to sub-pixel accuracy, $\rho_{M}$   ▹Equation (18)10:**end for**11:Calculate slope of locus at local maxima, $\rho_{M}^{\prime }$  ▹Equation (19)12:**for all** detected local maxima **do**13: Calculate image coordinates, (x,y)      ▹Equation (6)14:**end for**15:Link adjacent peaks into segments16:Link segments into complete peak locus    ▹Proximity in both parameter and image spaces17:Eliminate duplicate points in image space18:**while**min(cos(ϕ))<−0.5**do**       ▹ϕ is angle between adjacent pairs of pixels19: Remove corresponding outlier        ▹Boundary filtering20:**end while**21:Mark line between detected pairs of points

**Figure 20:**
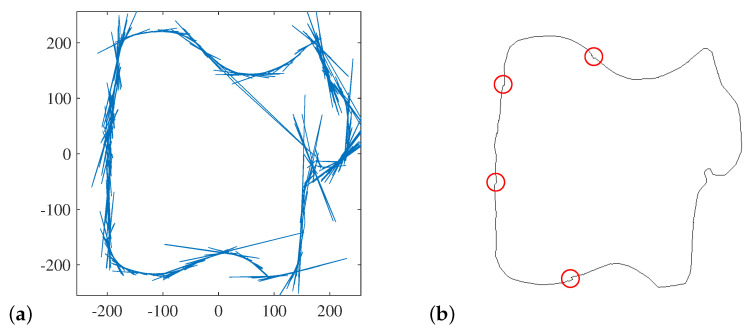
Filtered reconstruction of the boundary points. (**a**) Linked edge points (without filtering), clearly showing outliers. (**b**) After filtering successive points using a cosine similarity score; remaining significant noise on the boundary is highlighted.

The resulting peak locus is then filtered. First, there are several instances where adjacent points from the parameter peak locus map to the same image pixel. In these cases, all but one pixel is removed. Within the remaining sequence of pixels, one characteristic of outliers resulting from incorrect $\rho^{\prime }$ is a rapid change of direction between successive reconstruction points. This is clearly seen in Figure 20a, where the outliers are obvious. Let ϕ be the angle between successive pairs of reconstructed pixels. The cosine similarity metric is used to detect outliers, where cos(ϕ)≈−1 indicates that the boundary doubles back. Such pixels are removed in order of increasing cos(ϕ) to ensure that the most extreme outlier points are removed first. All boundary points where cos(ϕ)<−0.5 are removed. The result of this filtering is illustrated in Figure 20b. As a result of connecting the detected pixels, the boundary is now complete, and most of the significant outliers have been removed by the filtering process. There are a few minor noise points remaining on the boundary, but these are within two or three pixels of the original edge. The more significant of these noise points are indicated within Figure 20b. Note that filtering removed nearly 95% of the points reconstructed from the peak locus (from 4333 down to 253 pixels in this example). The remaining pixels, corresponding to approximately one in every four or five pixels along the boundary, are connected by straight lines. A single noise point can therefore result in triangular shaped artefacts such as seen in the top two highlighted areas in Figure 20b.

### 5.5. Inflection Points

**Figure 21:**
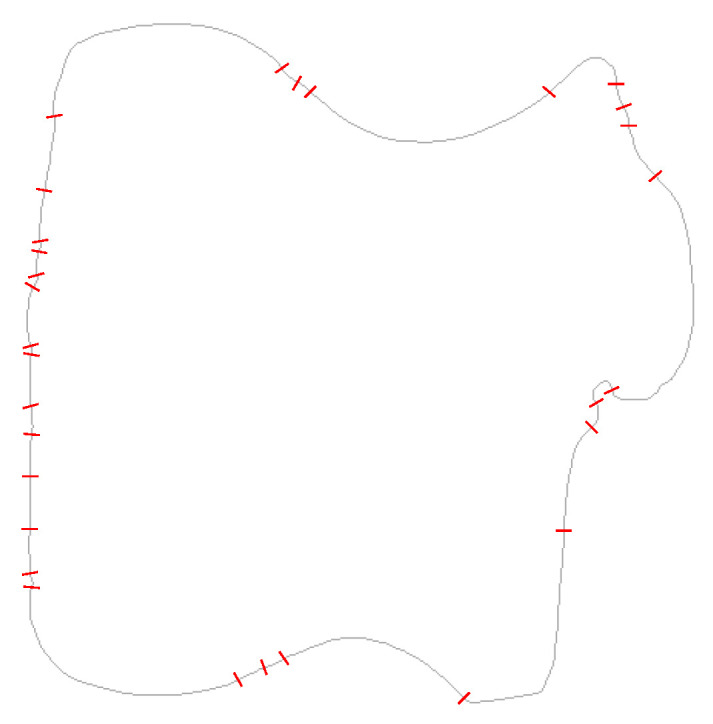
Inflection points marked on the original image, as detected from the Hough transform.

Inflection points are perhaps a little more reliable to detect because both edges of the cusp have the same angle. By detecting the cusps in the peak locus, these are then mapped back to the nearest edge point in image space to locate the inflections. The inflection points are marked in red in Figure 21. Visually, these clearly coincide with changes in sign of the curvature on the boundary, with many points detected on the rough left edge of the shape. The Hough transform is able to detect quite subtle variations in curvature. The angle difference is also amplified through the use of the gradient Hough transform, as these tend to extend the cusps by Δθ, exaggerating the angle. Note that in the full Hough transform (Figure 15) only the cusps associated with major concavities are visible, and even these are masked by the background clutter.

### 5.6. Convex Hull

**Figure 22:**
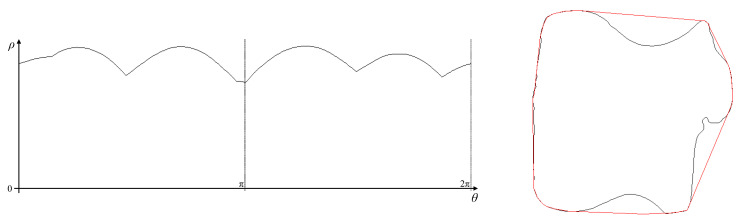
Convex hull, uses the outermost peak locus. The reconstructed convex hull is shown on the right superimposed in red on the original outline.

Finally, the convex hull of the shape can be reconstructed using Algorithm 6, with the result demonstrated in Figure 22. The detected peak locus with the maximum ρ for each angle is used (since it is a lighter object on a darker background). Linking of the locus points is trivial, because the convex hull has no boundary segments with negative curvature (by definition); the locus peaks are simply linked in order of increasing θ. The associated ρ and $\rho^{\prime }$ are used to reconstruct the boundary. As the reconstructed pixels contain outliers because of the uncertainty in the estimates of $\rho^{\prime }$, these are filtered using the same procedure as before (by removing pixels where the cosine similarity between adjacent pixels is cos(ϕ)<−0.5). The reconstructed boundary is very close to the convex hull of the original shape. Close inspection of the boundary does reveal one or two noise points (for example, on the bottom left corner where the convex hull goes slightly inside the original boundary, and the bottom boundary is slightly curved rather than straight), however these are very close to the original curve. Note that the curved sections of the locus are easier to reconstruct because an estimate of $\rho^{\prime }$ can be obtained more readily. The corners within the parameter locus either result from butterflies or the intersection of two parts of the locus; both of these reduce the accuracy of estimating $\rho^{\prime }$ and thus finding the endpoints of the linear segments. The linear segments of the convex hull are therefore formed from linking adjacent curved segments.
**Algorithm 6** Reconstruction of convex hull.**Input:** Vote image V[θ,ρ] from gradient Hough transform**Output:** Reconstructed image R[x,y]1:**for all**(x,y)**do**2: R[x,y]=0                  ▹Clear reconstruction image3:**end for**4:**for each**θ**do**5: Find topmost local maximum, $\rho_{m}$          ▹$V\left[\theta ,\rho_{m}\;-\;1\right]\leq V\left[\theta ,\rho_{m}\right]\geq V\left[\theta ,\rho_{m}\;+\;1\right]$6: **if** $V\left[\theta ,\rho_{m}\;-\;1\right]>V\left[\theta ,\rho_{m}\;+\;1\right]$ **then**7:  $\rho_{m}\leftarrow \rho_{m}-1$                ▹Peak is between $\rho_{m}$ and $\rho_{m}\;+\;1$8: **end if**9: Calculate peak to sub-pixel accuracy, $\rho_{M}$     ▹Equation (18)10:**end for**11:Calculate slope of locus at local maxima, $\rho_{M}^{\prime }$    ▹Equation (19)12:**for all** detected local maxima **do**13: Calculate image coordinates, (x,y)       ▹Equation (6)14:**end for**15:Link pixels in order of θ16:Eliminate duplicate points in image space17:**while**min(cos(ϕ))<−0.5**do**        ▹ϕ is angle between adjacent pairs of pixels18: Remove corresponding outlier          ▹Boundary filtering19:**end while**20:Mark line between detected pairs of points

### 5.7. Additional Examples

**Figure 23:**
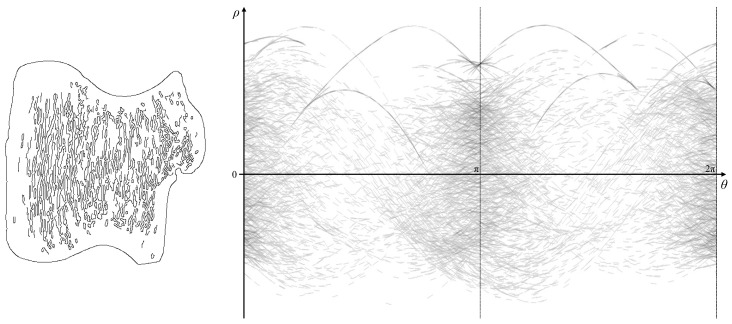
The shape with all edges present, and its corresponding gradient Hough transform (contrast enhanced for visibility).

The first example illustrates many of the features of the proposed approach. However, it consists only of a single curve comprising the outer boundary. The Canny filter not only detects the outer boundary, but also many internal edges. When incorporating these within the gradient Hough transform, the result is shown in Figure 23.

The dominance of vertical internal edgelets results in significant clutter within the parameter space around 0, π and 2π, with a scattering of voting trace fragments at other angles. Such clutter obscures many of the details of the parameter locus even of the outer boundary, making extraction of the locus difficult, if not impossible. The use of the gradient Hough transform results in many short trace fragments, making it impossible to link corresponding segments to reconstruct any internal detail. Note that the top edge of the parameter locus remains clearly visible, enabling reconstruction of the convex hull.

**Figure 24:**
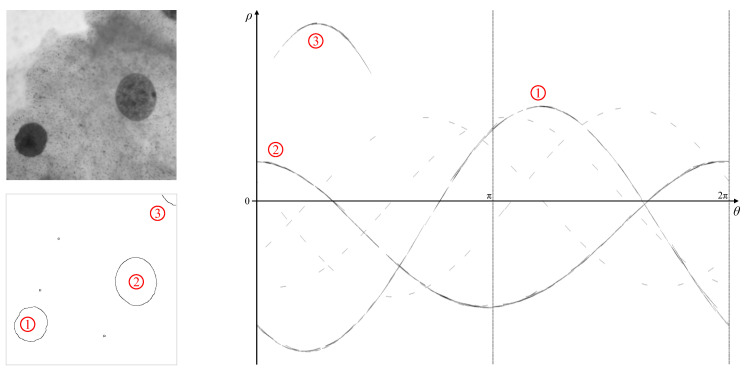
The shape with detected edges, and its corresponding gradient Hough transform (contrast enhanced for visibility).

A simpler example is shown in Figure 24. This 500×500 cell image was processed with a Canny filter with $\sigma =\sqrt{2}$ and thresholds [0.24,0.6] to detect two cells (labelled ➀ and ➁). A third partial cell ➂ and three background noise points were also detected. The complete cells result in two distinct, approximately sinusoidal, loci, with the incomplete cell giving only a partial locus. The noise points result in dashed parameter loci because the curvature of the boundary changes rapidly, and each boundary pixel only votes along a single dash with the gradient Hough transform.

**Figure 25:**
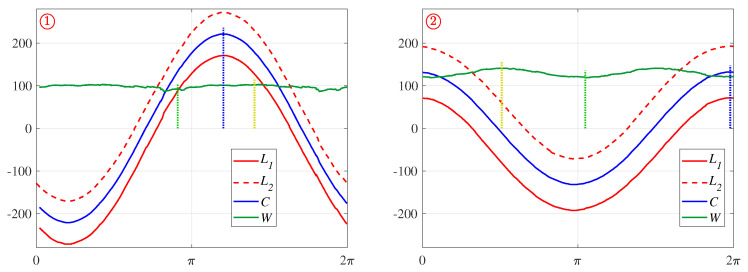
Using the parameter loci to derive parameters of the elliptical objects. The flipped locus, $L_{2}$ in Equation (9), centre line, *C* in Equation (11), and the Feret diameter, *W* in Equation (10), are shown for each of the two blobs. The blue, yellow and green dotted lines correspond to the detected feature points on the curves for object centre, major diameter and minor diameter, respectively.

Note that, because these are darker objects against a lighter background, any concavities show as features above the main locus rather than below. The parameter loci for the two complete objects have been extracted, and analysed for detecting the ellipse parameters as described in Section 4.1. The peak locus $L_{1}\left(\theta \right)$, inverted locus $L_{2}\left(\theta \right)$, along with C(θ) and W(θ) for each of the two objects are shown in Figure 25. The peak of C(θ) (the blue curve) gives the location of the centre of the object in the image, while the maximum and minimum of W(θ) (the green curve) give the lengths and orientations of the major and minor diameters, respectively.

The precise location of extrema of sinusoidal type curves is quite sensitive to any noise, because the curve is quite flat at the extrema. This is particularly noticeable for W(θ), especially for ➀, and simply finding the global extreme points can give significant errors, particularly in θ. Therefore, a parabolic curve is fitted to the points within 95% of the peak value for *C*, or $\theta_{peak}\pm \frac{\pi }{8}$ for *W*. The extreme point of the parabola then provides a more accurate estimate of the location and amplitude of the extreme of the underlying waveform. These smoothed locations are marked in Figure 25.

**Figure 26:**
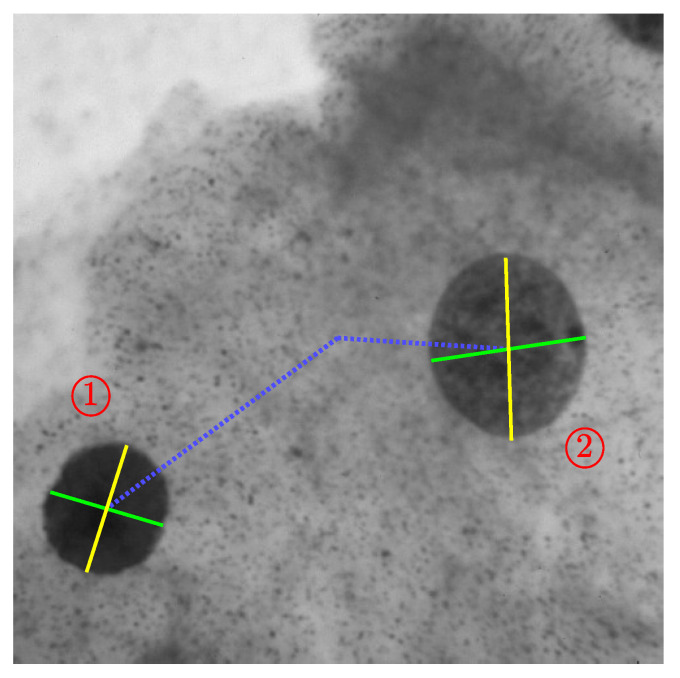
Detected elliptical objects. The blue line shows the object position relative to the image centre, with the yellow and green lines showing the major and minor diameters.

The detected features are overlaid on the original image in Figure 26. For object ➀, W(θ) is almost flat, indicating that the object is approximately circular. The major and minor diameters are 102 and 90 pixels, respectively. For object ➁, W(θ) is approximately sinusoidal, indicating a more ellipsoidal shape. The major and minor diameters are 140 and 120 pixels, respectively, showing greater eccentricity. However, the axes are not perpendicular (0.46π or 83.3°), indicating that this is not a true ellipse. Note also that for ➁ the detected centre is offset slightly, and the length of the major diameter is slightly shorter than the object. These are a result of noise giving a small angle error for the centre, and the parabolic smoothing shortening the diameter slightly.

## 6. Summary and Conclusions

This paper demonstrates that the line Hough transform has more utility than just detecting lines within an image. It is demonstrated that patterns within the parameter space of a line Hough transform can be used to identify features or characteristics of an arbitrary curve within image space. This has two advantages over other approaches for detecting arbitrary curves: First, parameter space is kept at two dimensions, which improves the efficiency of the transform. It is not necessary to go to higher dimensions to detect more complex curves. Second, it is truly capable of detecting arbitrary curves. The curves do not have to have parametric representations, or be restricted to a single shape (as required by the generalised Hough transform). As the underlying image features are generally not linear, the peaks within parameter space become spread out in a distinctive manner, resulting in a series of peak loci. Key features of the curve result in distinct patterns of these peak loci within parameter space, and it is the combination of these that characterise the curve.

To make the underlying patterns clearer, and reduce the clutter within parameter space, it is necessary to use a gradient Hough transform. This requires accurate detection of edgelets within an image, rather than just edge pixels, because each pixel has to have an associated orientation. By only voting along the tangent at an edge (a small range of angles is used to account for the uncertainty in orientation detection) only the key features are contributed to within the Hough transform, making the patterns easier to detect.

A significant contribution of this paper is therefore extending the duality within the Hough transform between lines and peaks to edglets and the corresponding peak loci. Such extension enables key features to be detected from the Hough transform such as:

- linear segments along the boundary (including dashed lines);
- inflection points (where the curvature changes sign);
- corners;
- orientation and ellipticity of elliptical shapes;
- the convex hull of the shape; and
- the depth of concavities.

The main strengths of the Hough transform for line detection are its robustness against noise and occlusions. Although the curve detected within the image is binary, it may be within an image with quite a lot of clutter. However, after the Hough transform, there is reinforcement from the superposition of multiple votes along the tangent of the curve, whereas any noise points will tend to vote randomly within parameter space, and not contribute to any significant features. The extent of this reinforcement is governed by the curvature of the edges of interest within the image. Regions of low curvature will have significant reinforcement from multiple edge points in the image voting for similar parameters. Such reinforcement gives robustness against noise (both from missing edge points and spurious votes from falsely detected pixels), making the locus easier to detect within parameter space than the original curve within image space. However, in areas of high curvature, this happens to a lesser extent, as the angle of the tangent changes more quickly from one pixel to the next. Points on a curve with high curvature will receive only a few votes on the locus, and there may even be gaps at corners. Such features are more sensitive to noise and occlusion of fine features cannot, in general, be recovered.

As with any use of the Hough transform, the algorithms presented in this paper require good detection of edges. This approach is more sensitive than detecting linear features because of the extended patterns patterns within parameter space. Use of the gradient Hough transform also requires the gradient to be estimated with reasonable accuracy. For complex shapes, the boundary can result in quite complex patterns, with the Hough transform becoming quite cluttered. This can make automatic detection of some of the key features difficult, particularly if they are aligned with other features, resulting in overlaps within the Hough transform.

To reconstruct the boundary of the object from the Hough transform, it is necessary to estimate the gradient of the peak locus. While tangents of the curve within image space can readily be reconstructed, specific points on that tangent require accurate estimation of the gradient. This is limited by the discretisation of parameter space, and will generally introduce noise within the reconstructed boundary. A filtering procedure has been described which can remove many of the obvious outliers, but still leaves some noise on the boundary.

### Future Work

The gradient Hough transform used here had a fixed voting window (±Δθ). One improvement would be to use a minimum Δθ, but to increase it adaptively based on local information within the image. This would avoid gaps within the locus where θ changes rapidly (as seen in Figure 16, for example at corners), while still reinforcing the locus where θ changes slowly.

It may also be worth considering filtering the θ estimates from the Canny filter. This could remove some of the spurious inflections resulting from noise on otherwise relatively straight edges. It would also enable Δθ to be reduced, reducing the blur of the peak locus. Associated with this, the Gaussian smoothing could be made adaptive, but this would require the design or selection of an appropriate control mechanism.

The method used to split the vote between adjacent pixels worked to some extent, but alternative approaches should be explored.

Alternative approaches for estimating the position of the peak locus need to be investigated, and in particular for determining the slope of the peak locus. The approach taken within this paper of linear interpolation to find the peak locus to sub-pixel accuracy is quite simplistic. Where the edge is curved, the peak locus will be along one edge of the region of votes, with the blur on the other edge increasing with increasing Δθ. If this model could be factored into the estimation of the peak locus position, then a more accurate locus may be found, especially where multiple loci intersect. The simple 3×3 window for estimating peak locus slope is adequate for isolated loci, but, when loci intersect, selecting the wrong local peak within a window gives spurious values for $\rho^{\prime }$ (outliers).

Another issue is the interaction between the gradient voting process; all values within ±Δθ are given equal weight. This can result in artifacts where the voting suddenly stops at the end of the window. Where the tangent is curved, the votes at the ends are less reliable, and can result in noise in estimating the peak locus position. While the effect of this is minor, these errors are amplified significantly when estimating $\rho^{\prime }$. One solution to this would be to taper the window with less weight at the ends, giving a smoother transition. Another approach to consider when estimating $\rho^{\prime }$ is to take into account more θ samples (rather than just one on either side) and use some form of low-order curve fit. This would both reduce noise, and provide a more accurate estimate of the slope.

Overall, this paper proposes a novel use of the Hough transform for analysing the shape of arbitrary curved shapes. It is shown that there is a one-to-one relationship between the shape of a curve, and its associated peak locus within Hough parameter space. This enables the shape to be analysed within parameter space, and the curve to be reconstructed directly from the peak locus.
